# Microbiome Analyses Demonstrate Specific Communities Within Five Shark Species

**DOI:** 10.3389/fmicb.2021.605285

**Published:** 2021-02-11

**Authors:** Rachael Storo, Cole Easson, Mahmood Shivji, Jose V. Lopez

**Affiliations:** ^1^Halmos College of Arts and Sciences, Nova Southeastern University, Fort Lauderdale, FL, United States; ^2^Department of Marine Sciences, University of Georgia, Athens, GA, United States; ^3^Biology Department, Middle Tennessee State University, Murfreesboro, TN, United States; ^4^Save Our Seas Foundation Shark Research Center, and Guy Harvey Research Institute, Fort Lauderdale, FL, United States

**Keywords:** microbiome, ecology, shark, microbial, holobiont, rRNA, richness, diversity

## Abstract

Profiles of symbiotic microbial communities (“microbiomes”) can provide insight into the natural history and ecology of their hosts. Using high throughput DNA sequencing of the 16S rRNA V4 region, microbiomes of five shark species in South Florida (nurse, lemon, sandbar, Caribbean reef, and tiger) have been characterized for the first time. The microbiomes show species specific microbiome composition, distinct from surrounding seawater. Shark anatomical location (gills, teeth, skin, cloaca) affected the diversity of microbiomes. An in-depth analysis of teeth communities revealed species specific microbial communities. For example, the genus *Haemophilus*, explained 7.0% of the differences of the teeth microbiomes of lemon and Caribbean reef sharks. Lemon shark teeth communities (*n = 11*) contained a high abundance of both *Vibrio* (10.8 ± 26.0%) and *Corynebacterium* (1.6 ± 5.1%), genera that can include human pathogenic taxa. The *Vibrio* (2.8 ± 6.34%) and *Kordia* (3.1 ± 6.0%) genera and *Salmonella enterica* (2.6 ± 6.4%) were the most abundant members of nurse shark teeth microbial communities. The *Vibrio* genus was highly represented in the sandbar shark (54.0 ± 46.0%) and tiger shark (5.8 ± 12.3%) teeth microbiomes. The prevalence of genera containing potential human pathogens could be informative in shark bite treatment protocols and future research to confirm or deny human pathogenicity. We conclude that South Florida sharks host species specific microbiomes that are distinct from their surrounding environment and vary due to differences in microbial community composition among shark species and diversity and composition among anatomical locations. Additionally, when considering the confounding effects of both species and location, microbial community diversity and composition varies.

## Introduction

Microbiomes help compose the “holobiont” or the total individual, which includes microbiota co-adapted with the macro-organismal host. Thus, microbiomes can contribute to organismal health through roles in the production of protective secondary metabolites (Nakatsuji et al., 2017; Rothschild et al., 2018), modulating host immunity (Apprill, 2017), or indicating a shift to a disease state (Caporaso et al., 2011; Llewellyn et al., 2014; Nelson et al., 2015; Sanders et al., 2015; Colston and Jackson, 2016; Lax et al., 2017). The term holobiont can refer to any sort of relationship between the microbial community and the host which is not simply limited to symbiotic relationships (Simon et al., 2019). While there is no direct support for holobiont coevolution in sharks, the general concept as shown in other organisms is worth considering (Zilber-Rosenberg and Rosenberg, 2008; Pita et al., 2018; Pratte et al., 2018; Freed et al., 2019; van der Loos et al., 2019; Carthey et al., 2020). Additionally, various anatomical parts within an individual organism can harbor significantly different microbiome communities. In humans, for example, individuals have significantly different microbiome compositions by anatomical location, likely stemming from a complex combination of host behavior, habitat, pH, diet, and varying life stage exposure to microbes (Human Microbiome Project Consortium, 2012a,b; Lloyd-Price et al., 2017; Carlson et al., 2018).

Sharks (class *Chondrichthyes;* subclass *Elasmobranchii*) spark biological interest for multiple reasons: sharks represent one of the oldest jawed vertebrate lineages dating back to over 450 million years, many species and populations are in decline due to overexploitation, they play important ecological roles as upper trophic level predators, and display intriguing biological features such as extreme longevity and the presence of molecular adaptations related to genome stability in some species (Dulvy et al., 2014; Nielsen et al., 2016; Marra et al., 2019). Despite the evolutionary antiquity, unique adaptations, and biological and ecological diversity of elasmobranchs, their microbiomes have received little attention with only a handful of studies published thus far (Apprill, 2017; Doane et al., 2017, 2020; Johny et al., 2018; Pogoreutz et al., 2019). A recent report found that the skin microbiome of the thresher shark (*Alopias vulpinus*) was significantly different and distinguished from the water column, while still hosting some taxa which were also represented in the surrounding water (Doane et al., 2017). In another interesting study, the microbiomes on the skin of four elasmobranch species, which are physiologically marked by dermal denticles and lower mucus secretions, had a higher phylosymbiotic signal than the skin microbiomes of teleost fishes (Doane et al., 2020). Pogoreutz et al. (2019) examined the microbiome of healthy and compromised skin on blacktip reef sharks (*Carcharhinus melanopterus*), finding significant community differences in individuals from different geographic areas, but not between healthy and injured skin on the same individuals. Johny et al. (2018) found nearly 25% of the gut microbiome of the deep-sea shark *Centroscyllium fabricii* was unable to be taxonomically classified at the phylum level, suggesting a large proportion of still uncharacterized microbial diversity in that shark microbiome.

Although rare, shark bites on humans do occasionally occur, with 64 unprovoked bites documented worldwide in 2019. The frequency of shark bites is higher in some regions of the world, including Florida, United States, due to large local human populations engaging in a high volume of recreational activities in the ocean (Florida Museum, 2020). Although not often fatal, shark bites can lead to an increased risk of bacterial infection, which are often treated with broad-spectrum antibiotics due to lack of pathogen knowledge (Unger et al., 2014). Characterizing the microbiome of shark teeth may help future bite treatment become more effective (e.g., vancomycin, fidaxomicin, and sarecycline) and targeted to narrower microbial groups.

Here, we provide a comparative assessment of the microbiome communities across four different anatomical sites (gills, teeth, skin, and cloaca) within each of five shark species common to South Florida waters (Florida Sea Grant, 2013): nurse (*Ginglymostoma cirratum*), lemon (*Negaprion brevirostris*), sandbar (*Carcharhinus plumbeus*), Caribbean reef (*Carcharhinus perezii*), and tiger (*Galeocerdo cuvier*) sharks. These five species occupy variable, but sometimes overlapping habitats (nearshore benthic to neritic waters, coral reef ecosystems, pelagic waters), and all have been implicated, at various levels, in human bite incidents. We tested three hypotheses about the microbiomes of these five shark species: (1) shark microbiomes are distinct from the surrounding seawater environment, (2) microbiomes vary in community composition among shark species, and (3) microbiomes of different anatomical locations within host species vary in microbial composition.

## Materials and Methods

### Sample Collection and Processing

Individuals of five shark species were caught off the southeast coast of Florida ranging from Boca Raton to Hollywood Beach at 9 locations (Supplementary Figure 18) and released once samples and measurements were taken. The sharks were caught using a drumline composed of a 22.68 kg weight and a line with a buoy on the top. Attached to the weight was a 30 m long line of 400 kg tested monofilament line with a circle hook and Atlantic bonito (*Sarda sarda*) as bait to attract sharks non-specifically. Fishing lines were set in groups of 10, with two lines at each of the following depths: 7.6, 12.2, 18.3, 24.4, and 30.5 m. Eight shark species were captured, but only five species had sufficient sample size (*n* ≥ 3) for downstream microbiome analyses. Microbiome samples of the gills, teeth, skin, and cloaca of each of the five shark species were taken using dual sterile swabs (Henry Schein, Melville, NY, Cat. 1228715). Swabs were placed in collection tubes in sealed bags and transported on ice to the laboratory. Experimental design followed the tenets of Knight et al. (2012), for the minimum number of samples required, as well as including all “metadata” associated with each sample.

Surface water was sampled into 10% HCl rinsed polypropylene bottles (Nalgene) once per sampling trip. Water samples were transported on ice to the laboratory for filtration through a 0.45 μl filter membrane immediately after each sampling trip so that environmental microbes could later be characterized. After filtration, environmental DNA (water samples) was extracted from the filter membranes using the DNeasy PowerLyzer PowerSoil kit (Cat# 12855-100), and swabs were extracted using the QIAamp BiOstic Bacteremia DNA kit (Cat# 12240-50) (MoBio Laboratories Inc.). DNA quality and quantity were checked using gel electrophoresis and Qubit and nanodrop before amplification. Extracted DNA was amplified using Polymerase Chain Reaction (PCR) with primers 806R and 515F FLXB targeting the V4 region of the 16S rRNA gene (Caporaso et al., 2011). Reactions contained 12.5 μL of AccuStart II PCR ToughMix, 9 μL of PCR grade water, 0.5 μL of each primer, and 2.5 μL of extracted DNA. Thermocycler protocol followed the Quantabio recommended protocol for the AccuStart II PCR ToughMix, with denaturation at 94°C for 3 min followed by 35 cycles of 30 s at 94°C, 30 s at 55°C, and 90 s at 72°C before holding at 4°C before processing. Sequencing of amplicons utilized an Illumina MiSeq sequencing platform equipped with a v2 chemistry 500 cycle cartridge (Caporaso et al., 2012). This yielded approximately 250 base pair paired-end sequences. All sequences were submitted to the Sequence Read Archive (SRA) under the projectaccession number: SRP111970 (Release date: 07-14-2017).

### Statistical Analysis

Initial processing of sequence data was performed in QIIME (Quantitative Insights into Microbial Ecology) version 1.9.1. Raw sequences were quality-filtered to remove all chimeric and low quality (quality score < Q30) sequences using the QIIME script split_libraries_fastq.py, with default settings which require each operational taxonomic unit (OTU) to contain at least two sequences. These sequences were then clustered into 97.0% similar OTUs using open reference clustering strategies utilized in the QIIME script pick_open_reference_otus.py (Caporaso et al., 2010). Taxonomic classification of OTUs was based on the SILVA database release 128 (Quast et al., 2013). OTUs found only in sea water (environmental samples) were excluded from the overall data set, as it was found that there was not a significant difference in the shark microbial communities with or without the included OTUs. All shark-associated OTUs were kept for downstream analysis, even if they only were found in one individual. Microbial community differences were examined between shark and environmental samples, among species, as well as among anatomical locations. Analysis was executed with the RStudio software (RStudio version 3.2.1), with the added libraries “*picante*” and “*vegan*” to compare the microbial diversity and composition of anatomical locations among species and in relation to the surrounding environment (Kembel et al., 2010; Oksanen et al., 2017).

Relative abundance of OTUs were calculated for use in downstream analysis in RStudio v. 3.2.1. Redundancy analysis with variation partitioning was performed to determine the amount of variance explained by host species and anatomical location using the *varpart* function in the *vegan* package (Oksanen et al., 2017). Levene’s test was used to assess homogeneity of variances and normality was tested with the Shapiro-Wilke test. Those comparisons which had significant *p*-values were submitted to the non-parametric Welch’s ANOVA and followed by a *post-hoc* Games-Howell test to examine significant differences between groups. For the comparisons where parametric testing was appropriate, statistical differences in richness, and Inverse Simpson diversity measure were assessed using an analysis of variance (ANOVA) (Oksanen et al., 2017), and Tukey’s HSD *post-hoc* tests were used to examine pairwise significant differences among groups.

Bray Curtis dissimilarities were calculated using the *vegdist* function of the *vegan* package (Oksanen et al., 2017). The *betadisper* function was used to assess homogeneity of variances of Bray-Curtis dissimilarities. Permuted multivariate ANOVA (PERMANOVA; *adonis* in *vegan* package) was used to assess significant differences in relative abundance of taxa among sample groups. Pairwise PERMANOVA was performed using the *EcolUtils* package in R with the preset metrics to assess which groups were significantly different in relative abundance of OTUs (Salazar, 2020). Next, an NMDS (Non-metric dimensional scaling) analysis was done on the calculated Bray-Curtis dissimilarities between groups to show differences in beta diversity of relative abundance of OTUs between samples. A SIMPER test (499 permutations) was then used to discriminate which specific microbial taxa distinguished groups based on the Bray-Curtis dissimilarities. SIMPER performed pairwise comparisons of data and estimated the average contributions of each sampling unit (OTU) to the overall dissimilarity between two groups at a time (Oksanen et al., 2017). SIMPER analysis was done using both shark species and anatomical location. For example, nurse sharks were compared to all other shark species and teeth were compared to all other anatomical location.

## Results

### Differences in Microbial Communities Between Environmental and Biological Samples

**FIGURE 1:**
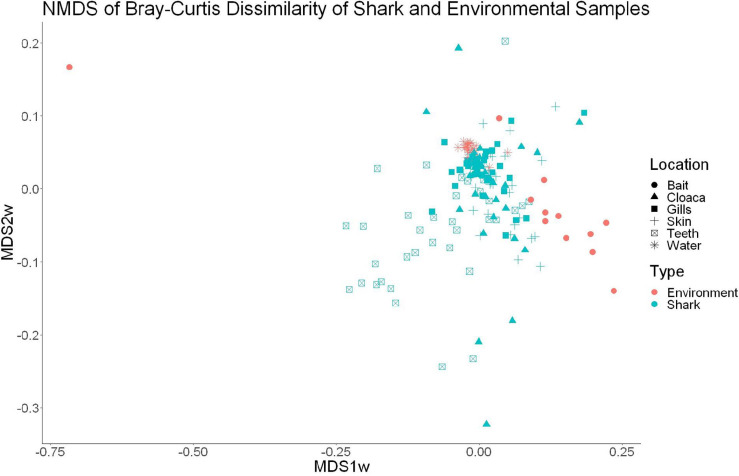
Non-metric dimensional scaling of shark and water samples (*R*^2^ = 0.77, stress = 0.1833).

High-throughput sequencing of 120 samples (gills, teeth, skin, and cloaca from each) from 30 sharks and 22 water samples which were taken once per trip produced a total of 12,374,571 high quality 16S rRNA reads, which resulted in 26,309 OTUs after QIIME processing. There were 25 OTUs found to be specific to water samples, which were later excluded from further analyses, leaving 26,284 that either were shark-specific or shared between water and shark samples. A 53.3 ± 24.6% overlap in OTUs appeared between all sharks and the surrounding water. Nonetheless, shark samples appeared significantly different from surrounding seawater as indicated by OTU richness (ANOVA, *df* = 1, *F* = 20.9, *p* = 9.23e^–5^) and diversity by the Inverse Simpson metric (ANOVA, *df* = 1, *F* = 10.36, *p* = 0.001). A PERMANOVA showed significant differences in community composition between shark and surrounding water samples (PERMANOVA, *df* = 1, *F* = 9.86, *R*^2^ = 0.05, *p* = 0.001). Non-metric dimensional scaling (*R*^2^ = 0.77, stress = 0.1833) demonstrates the similarities between surrounding water in shark microbial communities, which is consistent with shark-associated microbial communities that could be enriched from the water column. There is a clear outlier in this analysis, which could be due to contamination from handling of bait (Figure 1).

### Effects of Host on Microbial Diversity and Composition

**FIGURE 2:**
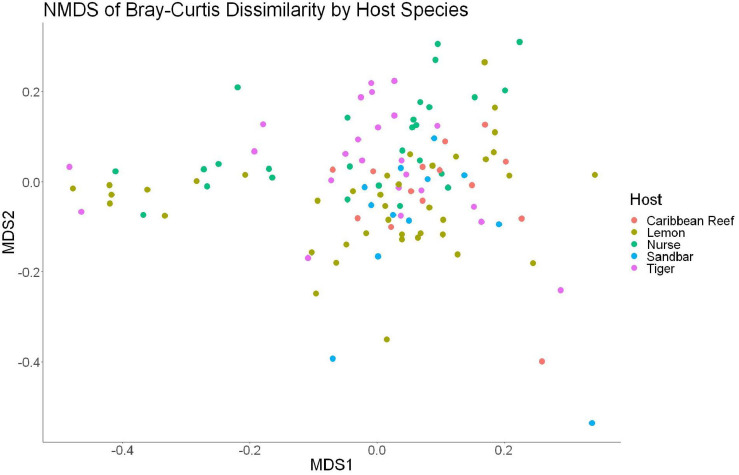
Non-metric dimensional scaling of all shark samples by shark species (*R*^2^ = 0.95, stress = 0.224).

The sample diversity when samples were grouped by shark species showed no significant differences in richness or diversity (Table 1) across host species. Host species is significant in explaining Bray-Curtis dissimilarity between samples (PERMANOVA, *df* = 4, *F* = 2.647, *R*^2^ = 0.104, *p* = 0.001). NMDS analysis (*R*^2^ = 0.95, stress = 0.224) showed some clustering by shark species but does not appear to fully explain differences in composition (Figure 2). A pairwise PERMANOVA revealed that all species were significantly different from one another by Bray-Curtis dissimilarity (i.e., all *p*-values were ≤ 0.02). Assessment of homogeneity of variances among sample groups indicated significant differences in the average distance to the spatial median among species which led to a non-parametric analysis (ANOVA, *df* = 4, *F* = 3.774, *p* = 0.006). Lemon and nurse sharks displayed significantly more variation among samples within their respective groups compared to other species. For example, lemon sharks which were sampled the most had the highest average distance to the median (0.6342), followed by nurse sharks (0.6221), tiger sharks (0.6096), sandbar sharks (0.5899), and Caribbean reef sharks (0.5832).

**TABLE 1:** Summary of statistics and sample size for each grouping considered in comparisons of the shark species.

Comparison	Sample size	Richness (ANOVA)	Inverse simpson (ANOVA)
Sharks + Water	117:25	*df* = 1, *F* = 20.9, *p* = 9.23e^–5^*	*df* = 1, *F* = 10.36, *p* = 0.001*
Species	117	*df* = 4, Welch’s *F* = 2.08, *p* = 0.102	*df* = 4, Welch’s *F* = 1.48, *p* = 0.225
Anatomical location	117	*df* = 12, *F* = 1.323, *p* = 0.22	*df* = 3, Welch’s *F* = 6.49, *p* = 0.000693*
Species * Anatomical location	117	*df* = 3, *F* = 1.292, *p* = 0.236	*df* = 3, Welch’s *F* = 10.0, *p* = 0.000019*
Teeth * Species	29	*df* = 4, *F* = 2.998, *p* = 0.04*	*df* = 4, *F* = 5.148, *p* = 0.004*

* in the comparison column indicates analysis based on the combination of variables.

### Effects of Anatomical Sample Site on Microbial Diversity and Composition

**FIGURE 3:**
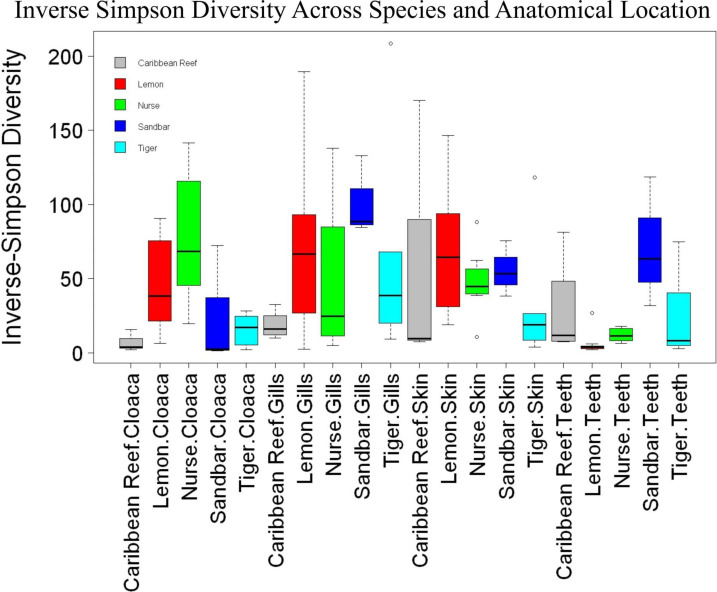
Box plot of mean species diversity of anatomical locations within shark species by the Inverse Simpson index (ANOVA, *df* = 117, Welch’s *F* = 5.01, *p* = 0.000113).

**FIGURE 4:**
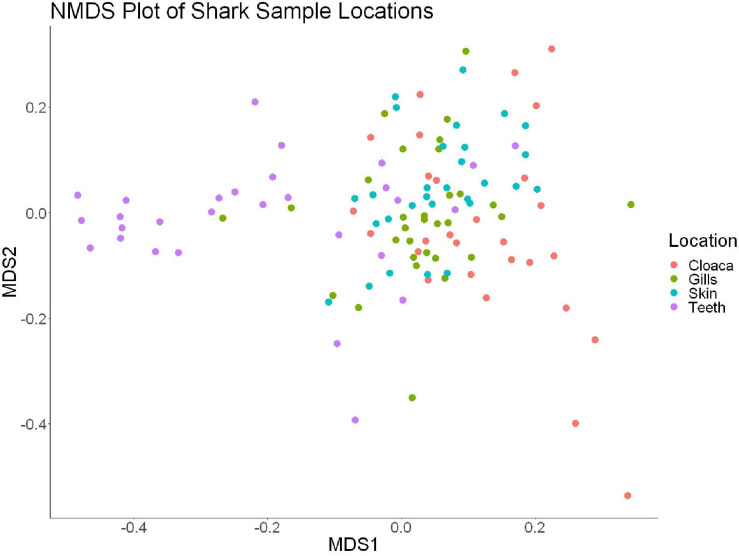
Non-metric dimensional scaling of all shark samples by anatomical location (*R*^2^ = 0.95, stress = 0.224).

**FIGURE 5:**
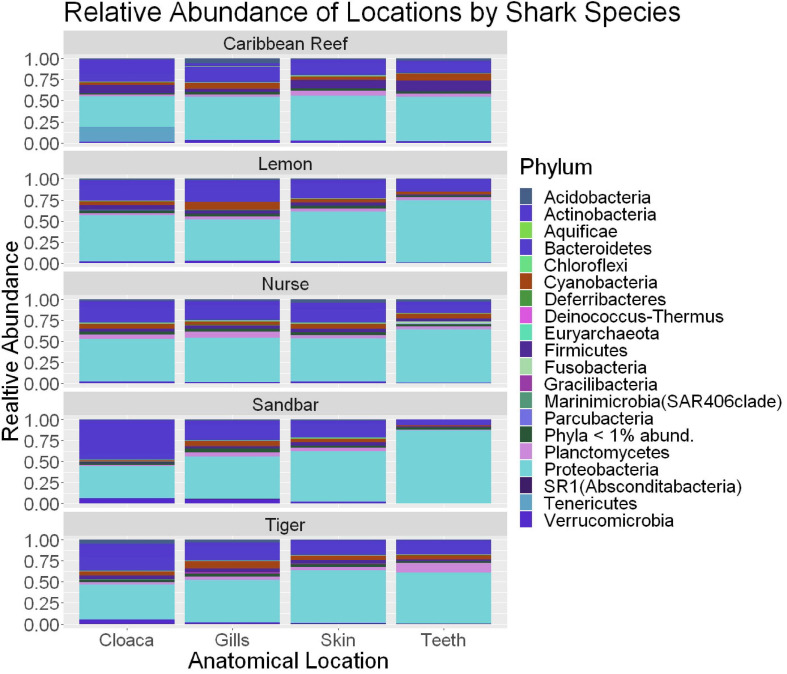
Stacked bar plot showing the relative abundance of phyla for shark species by anatomical locations (cloaca, gills, skin, teeth). All shark-associated samples and teeth-associated samples are represented here.

We observed significant differences in the Inverse Simpson’s diversity between anatomical sampling locations within each species (ANOVA, *df* = 3, Welch’s *F* = 6.49, *p* = 0.0007) (Table 1) with microbial diversity from teeth samples being significantly less than gill (Games-Howell *p* = 0.01) and skin (Games-Howell *p* = 0.01) samples. Significant differences were not found in microbial community richness among anatomical locations (Table 1). Differences were observed in diversity (ANOVA, *df* = 117, Welch’s *F* = 5.01, *p* = 0.000113) (Figure 3) but not richness (Table 1) when considering both host species and anatomical location. Variation partitioning revealed that variability is largely unexplained by just species and anatomical location for both richness (96%), diversity (89%), and composition (90%). Species was less important than anatomical location in explaining variability in richness (9 vs. 1%), diversity (9 vs. 1%), and composition (5 vs. 4%). Significant compositional differences by anatomical location were observed (PERMANOVA, *df* = 3, *F* = 2.12, *R*^2^ = 0.104, *p* = 0.001), and appear to be driven by teeth communities (Table 2.) NMDS analysis (*R*^2^ = 0.95, stress = 0.224) showed that while most samples cluster similarly, the teeth and cloacal samples separate out (Figure 4). A pairwise PERMANOVA showed that all anatomical locations were significantly different from one another by Bray-Curtis Dissimilarity (i.e., all *p*-values were ≤ 0.02) except for the cloaca and skin, which were not significantly different (*p* = 0.12). Microbial communities in association with anatomical locations were overall due to variations in the *Proteobacteria* phylum and notable shifts in *Actinobacteria* (Figure 5). For example, Simper results show that 9.4% of differences between skin and cloaca sample microbial compositions are explained by *Solirubrobacterales*. Additionally, *Sphingomonadales* explain 14% of differences between skin and teeth and 13% between cloaca and teeth samples.

**TABLE 2:** Summary of Pairwise PERMANOVA results for Bray Curtis dissimilarity by anatomical location (99 permutations).

	Cloaca	Gills	Skin
Gills	*p* = 0.012	–	–
Skin	*p* = 0.012	*p* = 0.060	–
Teeth	*p* = 0.012	*p* = 0.012	*p* = 0.012

**FIGURE 6:**
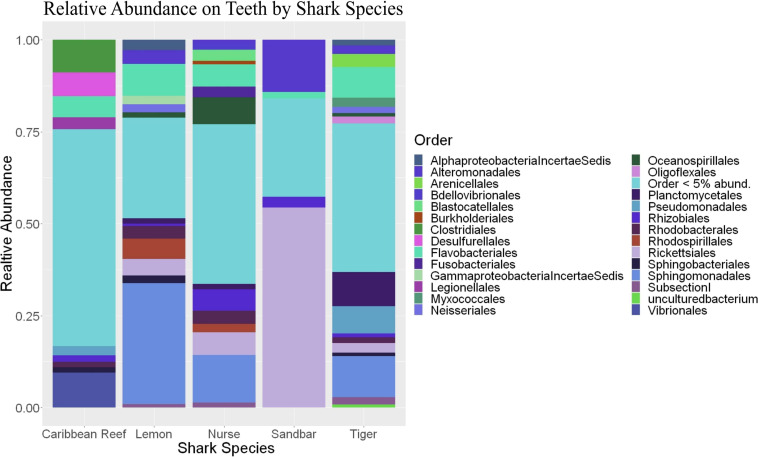
Stacked bar plot showing the relative abundance of phyla for teeth by shark species.

**FIGURE 7:**
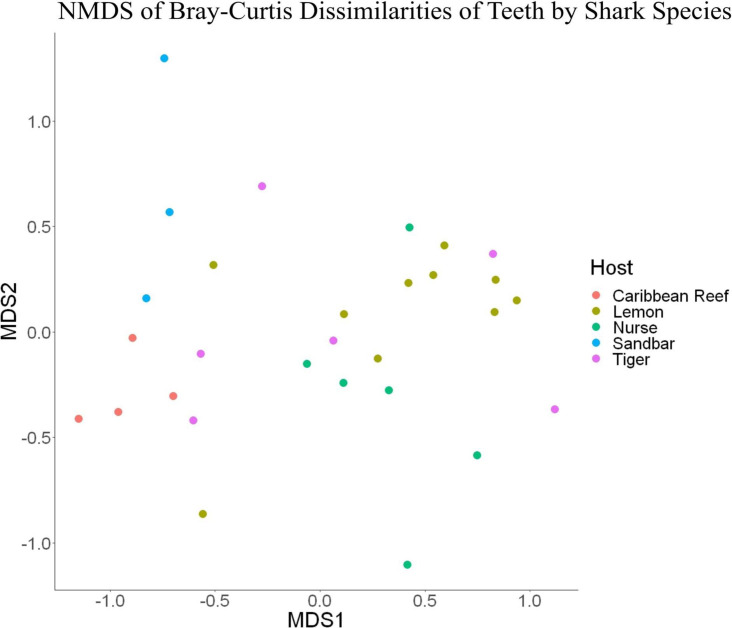
Non-metric dimensional scaling of teeth samples by shark species (*R*^2^ = 0.95, stress = 0.224).

Because teeth microbial communities had significantly less diversity than the gill and skin microbiomes and are one of the main drivers in difference by anatomical location (Table 2), we performed further analyses of teeth microbial communities among the five shark species and found them to be significantly different by species. Differences in microbial community richness in teeth samples of all species existed (ANOVA, *df* = 4, *F* = 2.998, *p* = 0.0377), as well as diversity, measured by the Inverse Simpson index (ANOVA, *df* = 4, *F* = 5.148, *p* = 0.0036). Significant differences in teeth community richness were mostly driven by the differences between lemon and Caribbean reef shark teeth (Tukey’s HSD, *p* = 0.024), with lemon sharks exhibiting a higher richness than Caribbean reef sharks. This is reflected in the relative abundance at the order level, which notably shows that lemon sharks have far higher relative abundance of *Sphingomonadales* compared to Caribbean reef sharks. In addition, Caribbean reef sharks host the largest relative abundance of *Vibrionales* (Figure 6). Significant differences in teeth microbiome compositions by host species were observed (PERMANOVA, *df* = 4, *F* = 2.7139, *R*^2^ = 0.303, *p* = 0.001). Caribbean reef and sandbar shark teeth samples appear most different from lemon and nurse (NMDS, *R*^2^ = 0.95, stress = 0.224) (Figure 7). Pairwise comparisons of teeth samples by shark species revealed that all species have significantly different compositions except when comparing sandbar and Caribbean reef sharks (Table 3). Microbial communities appear to be species-specific based on these results outlining significant difference by host species.

**TABLE 3:** Summary of Pairwise PERMANOVA results for Bray Curtis dissimilarity of teeth samples by shark species (99 permutations).

	Caribbean reef	Lemon	Nurse	Sandbar
Lemon	*p* = 0.037	–	–	–
Nurse	*p* = 0.037	*p* = 0.033	–	–
Sandbar	*p* = 0.037	*p* = 0.033	*p* = 0.040	–
Tiger	*p* = 0.037	*p* = 0.037	*p* = 0.037	*p* = 0.040

The most abundant taxa in shark teeth microbial communities are depicted in Supplementary Figures 9–13 and show several taxa that are in groups that contain human pathogens. For example, *Vibrio* spp., did not occur within the 10 most prevalent taxa in all teeth and varied widely in relative abundance by species. The *Vibrio* genus was represented in the sandbar (54.0 ± 46.0%), tiger (5.8 ± 12.3%), nurse (2.8 ± 6.34%), and lemon shark (10.8 ± 26.0%) teeth microbiomes. This taxonomic group, or any other potentially pathogenic group, was not found to be among the most abundantly represented in the surrounding water (Supplementary Figure 14). SIMPER results comparing lemon to Caribbean reef sharks also revealed OTUs which drive the differences between the teeth microbiomes in these two species. For example, the genus *Haemophilus*, explained 7.0% of the differences of the teeth microbiomes of these species. The *Haemophilus* genus includes some pathogenic species, such as *Haemophilus influenzae*, but members in this genus have also been associated with the saliva microbiome in humans in a non-pathogenic setting. When examining the SIMPER comparison between nurse and lemon shark teeth, the genus *Kordia* explained about 2.3% of differences.

## Discussion

In this study, we found that shark-associated microbial communities were distinct from the surrounding seawater and were significantly different among the species sampled. Previous studies indicate that holobiont microbiomes significantly differ from surrounding non-symbiont communities—e.g., seawater (Thompson et al., 2017; Freed et al., 2019). Based on our findings, some microbial taxa clearly overlap between the microbial community of the surrounding seawater and host shark microbial communities. We showed that overall microbial community composition was significantly different between shark species. Additionally, we found that anatomical location microbiomes varied significantly in composition when comparing between shark species.

Previous work has shown the presence of a community which co-evolves with the host in other organisms and systems (Zilber-Rosenberg and Rosenberg, 2008; Pita et al., 2018; Pratte et al., 2018; Freed et al., 2019; van der Loos et al., 2019; Carthey et al., 2020). Our findings support specialized microbial communities partially explained by anatomical location and shark species. Different shark species could inhabit niches or exhibit behaviors which could be related to the differential microbial communities characterized here. Environmental influences on natural microbial communities could include pollutants, migration patterns, pH, and salinity. For example, sharks are known to inhabit depths of 90 m or deeper during migration but move to shallower waters for parturition (Beck, 2016). The frequency of this behavior could contribute to the overall microbiome community composition sharks, as environmental factors could change with water depth. For example, tiger sharks have a proposed gestation period of 12 months (Castro, 2009), while nurse sharks have a much shorter 5–6 month long gestation period (Castro, 2000). Earlier environmental and host associated microbiome profiles of S. Florida, which our laboratory has been systematically characterizing, may add to the more routinely monitored environmental parameters (Thomas et al., 2016; Freed et al., 2019).

When comparing samples from all anatomical location for differences by shark species, there was no significant difference in overall microbial community diversity or richness (Table 1 and Figure 3). There was a significant difference in the composition by shark species, suggesting that there are factors which this study did not account for in addition to species which are contributing to unique communities. Our data support this, with variance partitioning showing that richness (95.7%), diversity (89.4%), and composition (89.7%) have substantial residuals which could in part be explained by environmental differences such as pollution, temperature, pH, etc.

When microbial communities of anatomical locations while also considering shark species were compared across all samples, there was a significant difference in the diversity of these communities, but not in richness (Table 1 and Figure 3). Community composition was also significantly different between anatomical location when considering host species. Our analyses found that only 5.2% of the variability in richness is explained by host species and anatomical location, suggesting that other environmental factors have important effects on richness of shark-associated microbial communities. Additionally, 10.7% of variability in diversity and 10.3% in composition are explained by host species and anatomical location. When considering richness (9 vs. 1%), diversity (9 vs. 1%), and composition (5 vs. 4%) anatomical location is the main driver, suggesting associated specialized microbial niches. This further emphasizes that these shark-associated communities are heavily dependent upon other environmental and perhaps behavioral factors which would allow for specialized niches.

Distinct microbial communities on shark teeth which are less diverse than other anatomical locations studied were drivers for compositional differences among anatomical locations based on pairwise comparisons (Table 2), leading to a more in-depth examination of the teeth microbial communities between host species. Previous studies show species, individual, or ethnicity specific microbial communities (Mason et al., 2013; Hernandez-Agreda et al., 2018; Ribeiro and Arnold, 2019). We showed that shark teeth host species-specific microbiomes which are unique in diversity, richness, and composition (Figure 7). It has been shown that differences in diet can result in differential oral microbiomes in other organisms (Adler et al., 2016; Fan et al., 2018), but this issue has been minimally explored in sharks. It is possible that differential diets effect the teeth microbiomes of sharks. For example, nurse sharks (*Ginglymostoma cirratum*) which are primarily benthic dwellers, frequently sedentary, and show high site fidelity (Castro, 2000), while tiger sharks (*Galeocerdo cuvier*), which occupy the mid to surface of the water column and often migrate vast distances (Lea et al., 2015). Tiger sharks also have highly cosmopolitan diets which include bivalves, teleosts, reptiles, and mammals and show ontogenetic variability (Dicken et al., 2017). However, dietary habits are not well-characterized for all shark species and can vary by individual and geographically (Simpfendorfer et al., 2001), so it is difficult to make conclusions about teeth microbial communities based only on host species.

With a larger sample size and more data, teeth microbiomes could reveal microbial taxa which could serve as biomarkers to identify shark species involved in a bite incident, or to improve bite treatment, which would be useful for forensics and ecological contexts. Potential infections after any deep bite wound are of concern. However, extensive profiling of the oral microbiome has occurred mostly for humans, with fewer current studies of other animals (Roggenbuck et al., 2014; Rojas et al., 2020). Bacteria isolated from infected bite wounds can often reflect the oral flora of the organism responsible for the bite, as exhibited in fish, mammals, reptiles, and birds (von Graevenitz et al., 2000; Abrahamian and Goldstein, 2011). Several microbial taxa found to be associated with shark teeth in this study belong to taxonomic groups that have been causes for concern in other animal bite wounds, such as *Streptococcus*, *Staphylococcus*, *Corynebacterium*, *Enterococcus*, and *Haemophilus*. The taxa *Vibrio*, *Salmonella enterica*, *Psychrobacter*, and *Halomonas* were also found, and are mostly associated with bites from aquatic organisms and reptiles. *Vibrio* has previously been found to be a concern predominantly in shark bites (Abrahamian and Goldstein, 2011).

*Vibrio* has been cultured previously from shark teeth, with *Vibrio parahaemolyticus*, *Vibrio alginolyticus*, and *Vibrio fluvialis* being present in a beached white shark’s teeth (*Carcharodon carcharias*) (Buck et al., 1984). Additionally, *Vibrio carchariae* has previously been isolated from an infected shark bite wound in a human victim (Pavia et al., 1989). Wounds from another two infected shark bites were sampled and cultured to show *Vibrio parahaemolyticus* in one patient and *Vibrio alginolyticus* in the other (Royle et al., 1997). Bite wounds are known to include bacteria which are found on the skin of the victim or in the surrounding environment (Abrahamian and Goldstein, 2011), but our data shows that V*ibrio* was not found in high relative abundance in the waters sampled in the vicinity of the shark captures.

## Conclusion

South Florida sharks host bacterial microbiomes distinct from the surrounding seawater environment and differ among taxa in microbial community richness and diversity. Microbiomes by host species appear driven by differences in composition, while microbial communities compared by anatomical sample locations are driven by the diversity and composition of the community. Host-associated microbial communities vary in composition when considering both anatomical location and host species. Because we show that teeth microbial communities are dependent upon host species, data generated in this study could be applicable to improvement in shark bite treatment and could serve as a biomarker to identify shark species involved in a bite incident which would be useful for ecological context. Future research should focus on bacteria found in shark teeth to determine if those strains present are truly pathogenic (following Koch’s postulates or carry viable virulence markers, etc.) to provide tangible insights to bite treatment or for future use as biomarkers for identifying shark species involved in bite events.

## Data Availability Statement

The datasets generated for this study can be found in the online repositories. The names of the repository/repositories and accession number(s) can be found in the article/Supplementary Material.

## Ethics Statement

The animal study was reviewed and approved by the Nova Southeastern University Animal Care and Use Committee, IACUC Protocol 2017.11.MS1.

## Author Contributions

RS performed the experimental methods and the statistical analysis with assistance from CE. RS wrote the first draft of the manuscript. All authors contributed to the conception and design of the study, contributed to the manuscript revisions, read, and approved the submitted version.

## Conflict of Interest

The authors declare that the research was conducted in the absence of any commercial or financial relationships that could be construed as a potential conflict of interest.
