# Supplementary material for: Microbiome Analyses Demonstrate Specific Communities Within Five Shark Species
Source: Front Microbiol. 2021 Feb 11;12:605285. doi: 10.3389/fmicb.2021.605285 (PMC7904884; doi:10.3389/fmicb.2021.605285)
Supplement: Supplementary file 1 [file Data_Sheet_1.doc]

**II. Supplemental Figures:
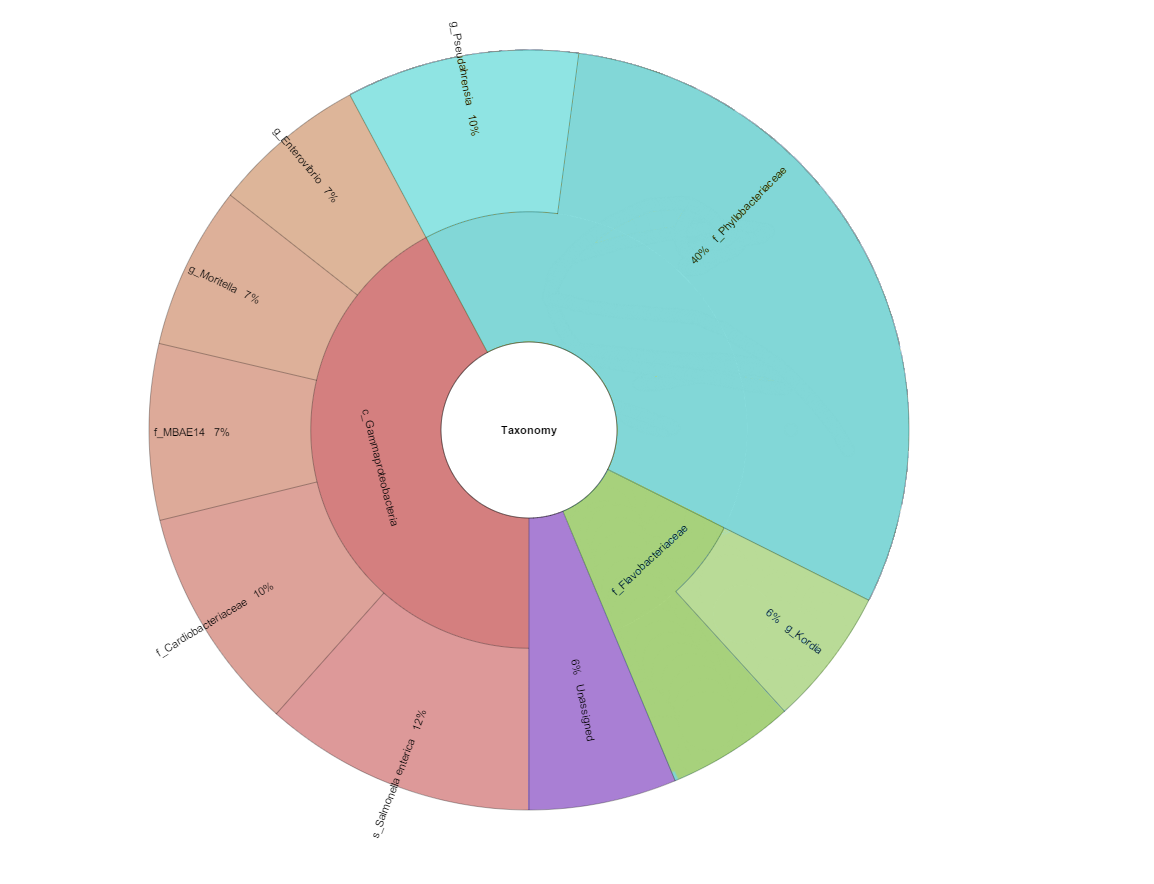
**

**Figure S1.** Krona Graphical representation of the 10 most abundant taxa in all sample locations of the nurse sharks sampled. Percentages are calculated based on overall relative abundance across all nurse sharks.


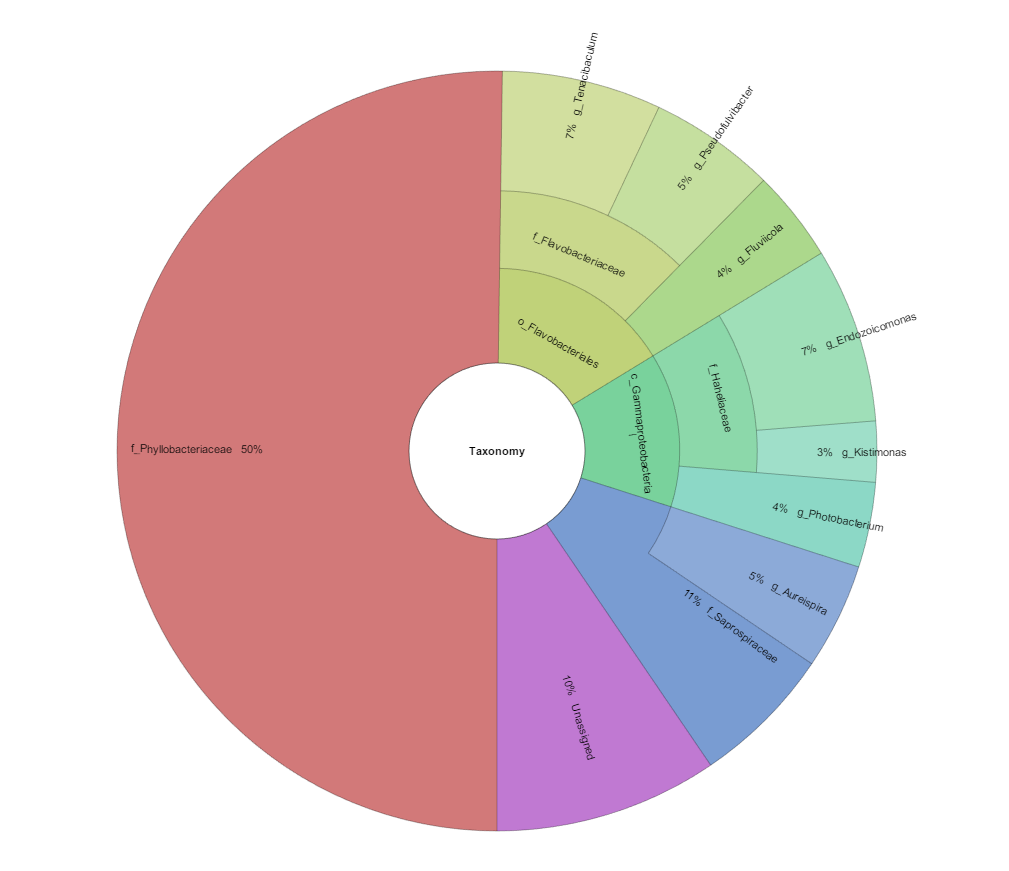


**Figure S2.** Krona Graphical representation of the 10 most abundant taxa in all sample locations of the lemon sharks sampled. Percentages are calculated based on overall relative abundance across all lemon sharks.


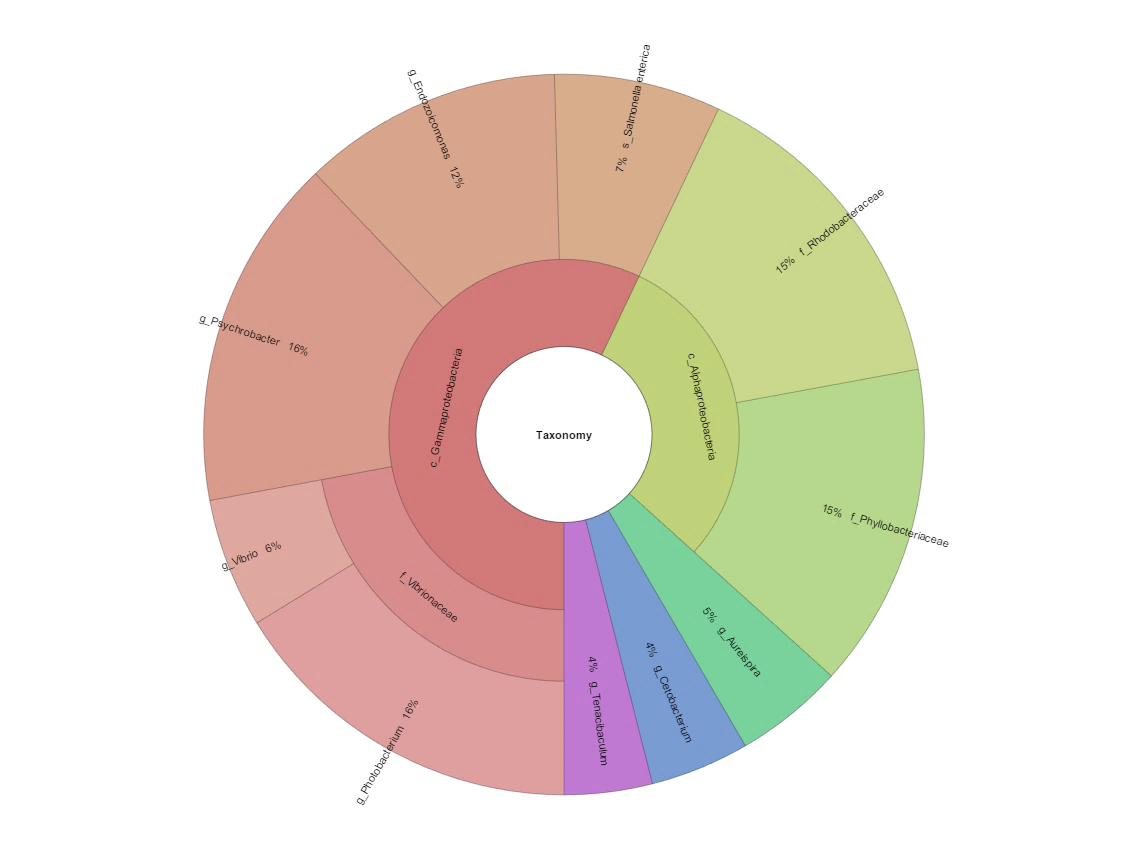


**Figure S3.**  Krona Graphical representation of the 10 most abundant taxa in all sample locations of the tiger sharks sampled. Percentages are calculated based on overall relative abundance across all tiger sharks.


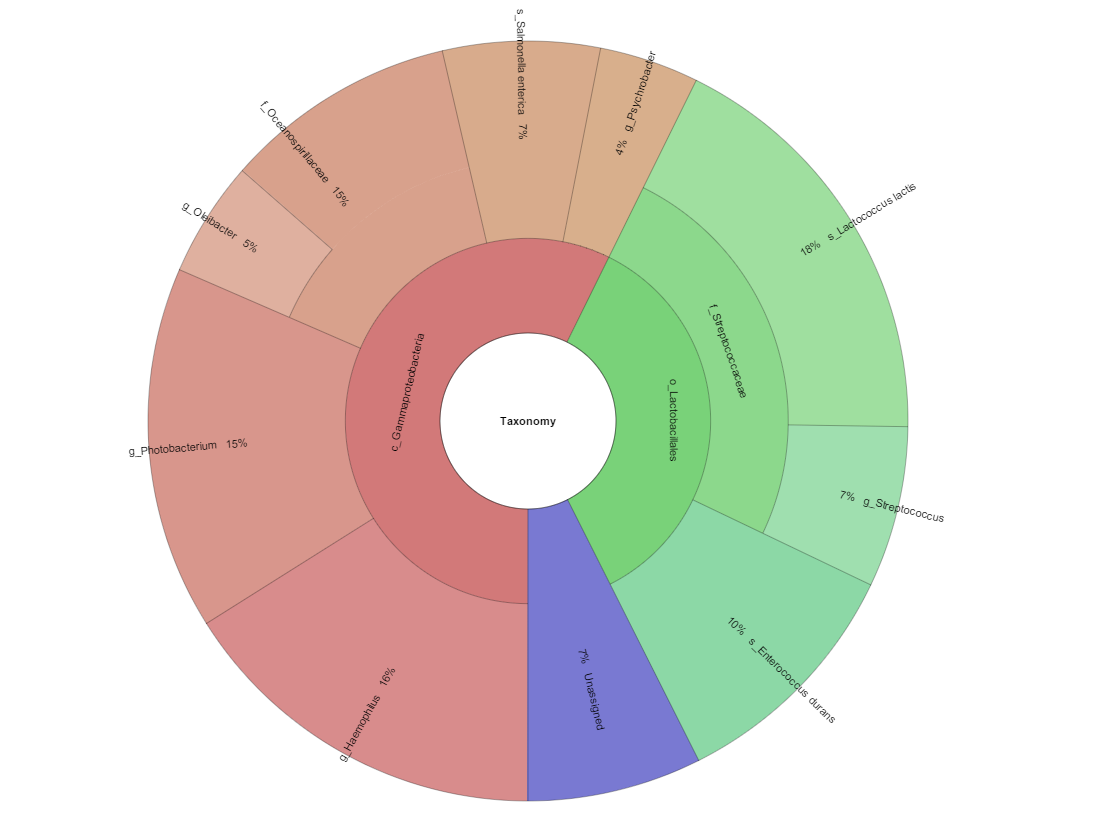


**Figure S4.**  Krona Graphical representation of the 10 most abundant taxa in all sample locations of the Caribbean reef sharks sampled. Percentages are calculated based on overall relative abundance across all Caribbean reef sharks.


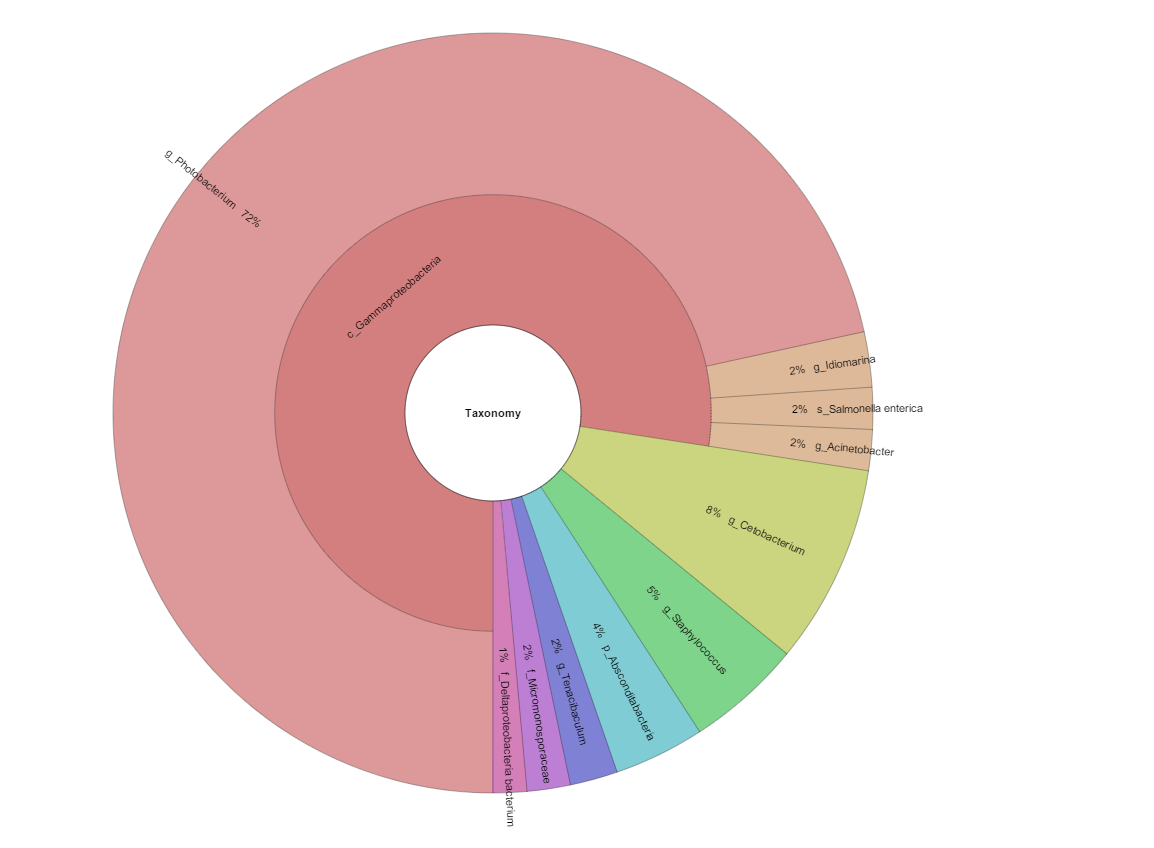


**Figure S5.**  Krona Graphical representation of the 10 most abundant taxa in all sample locations of the sandbar sharks sampled. Percentages are calculated based on overall relative abundance across all sandbar sharks.


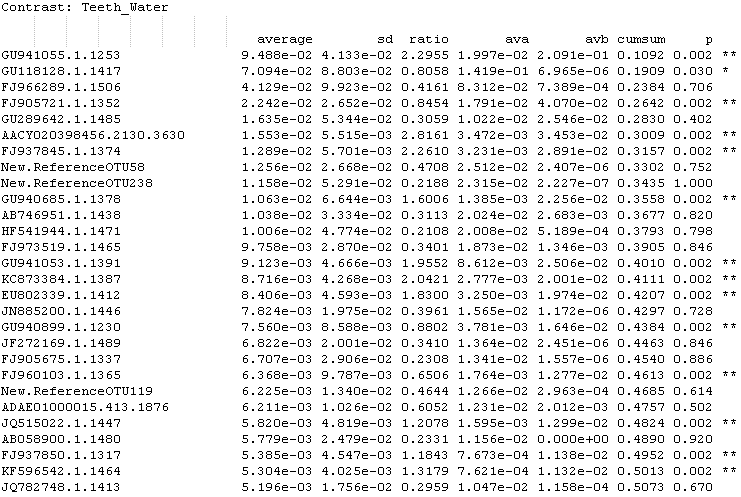


**Figure S6.** Simper analysis comparing all teeth to water sample OTUS, up to a cumulative sum of .5 (50.0%). (Significant codes: *= .05 **=.01)


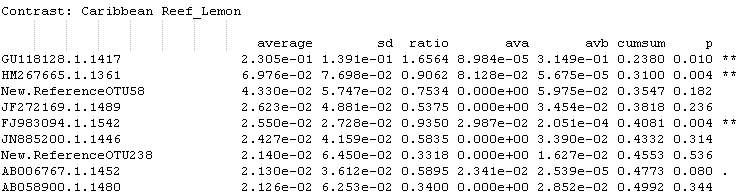


**Figure S7.** Simper analysis comparing all Caribbean reef to lemon shark teeth sample OTUS, up to a cumulative sum of .5 (50%). (Significant codes: *= .05 **=.01 ‘.’=.5)


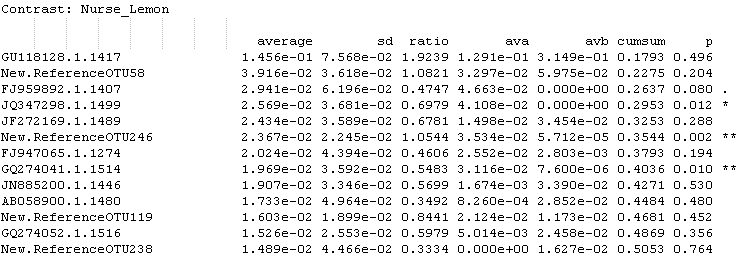


**Figure S8.** Simper analysis comparing all nurse to lemon shark teeth sample OTUS, up to a cumulative sum of .5 (50.0%). (Significant codes: *= .05 **=.01 ‘.’=.5)


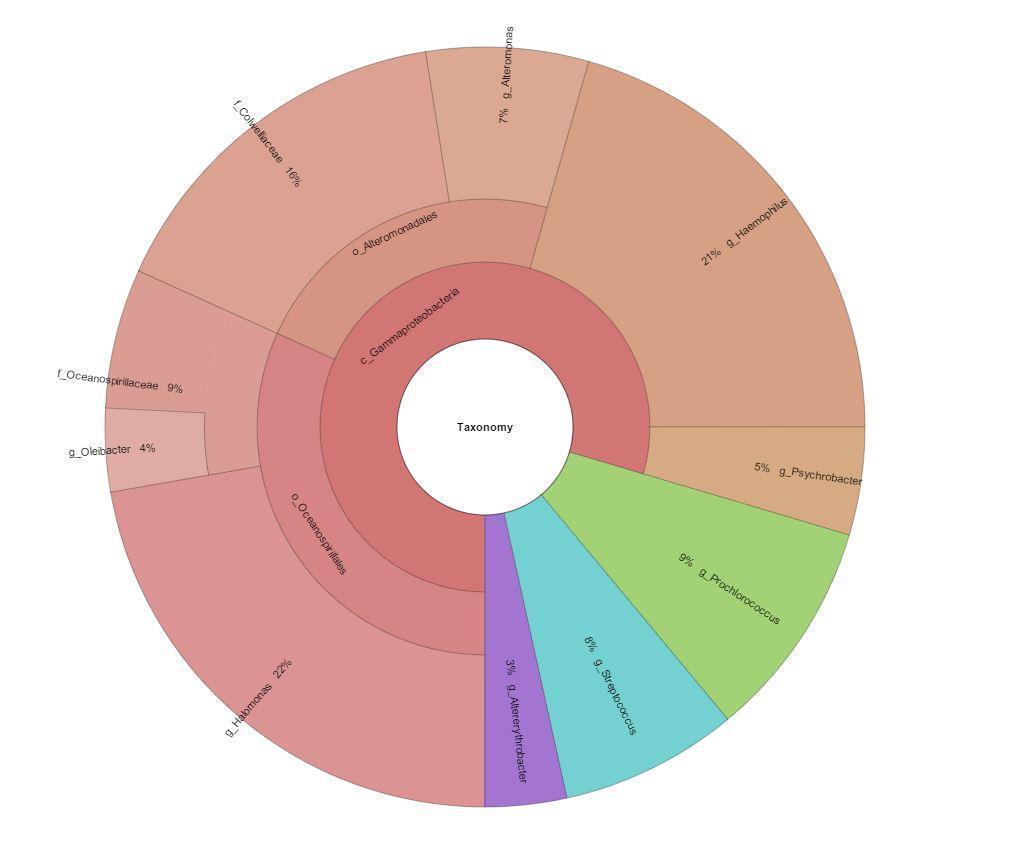


**Figure S9.**  Krona Graphical representation of the most abundant taxa in the microbial community of the teeth of the Caribbean reef sharks sampled. Percentages are calculated based on overall relative abundance across all Caribbean reef shark teeth samples.


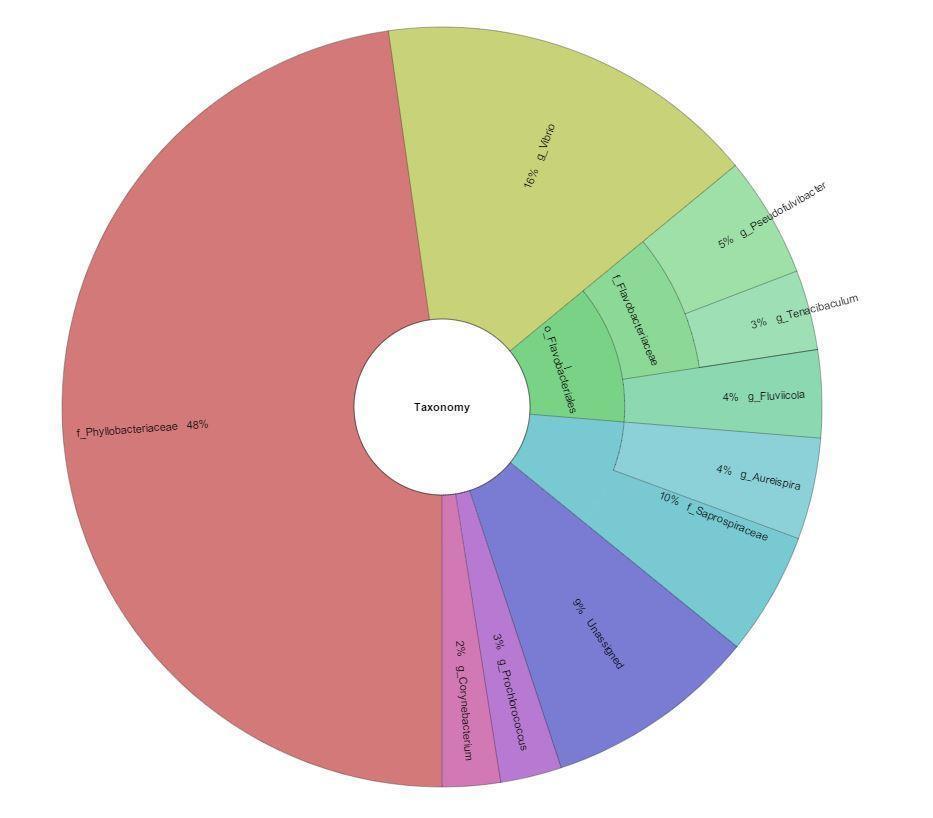


**Figure S10.**  Krona Graphical representation of the most abundant taxa in the microbial community of the teeth of the lemon sharks sampled. Percentages are calculated based on overall relative abundance across all lemon shark teeth samples.


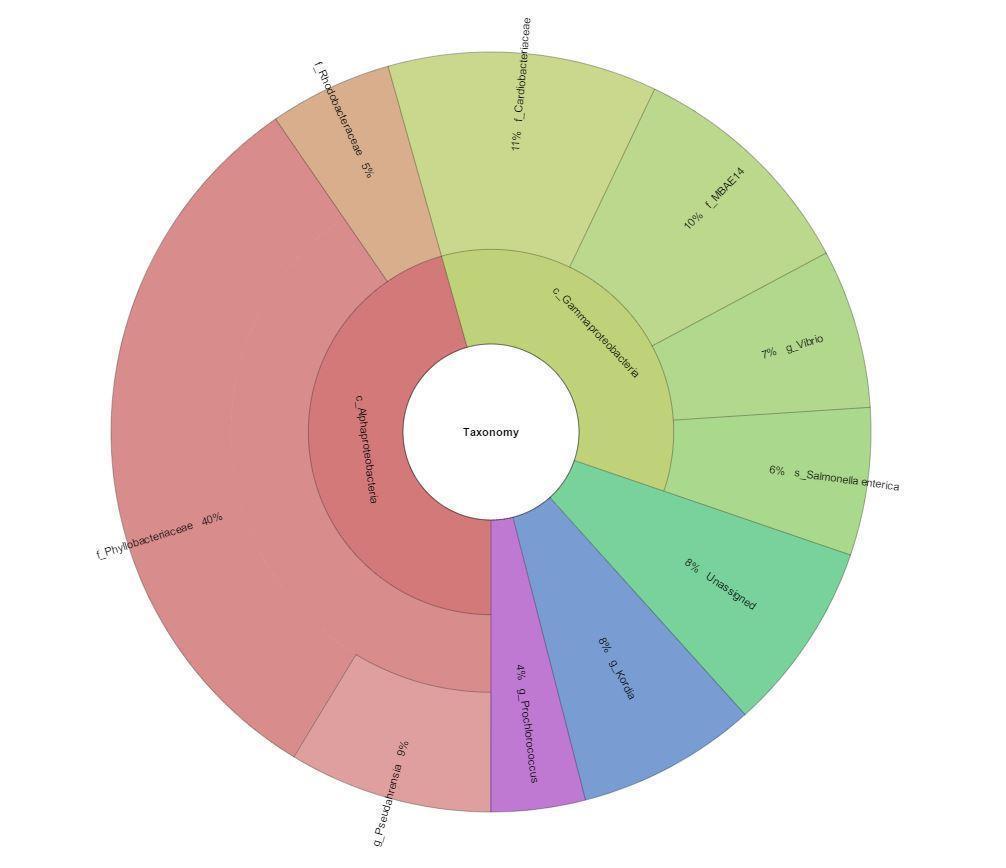


**Figure S11.**  Krona Graphical representation of the most abundant taxa in the microbial community of the teeth of the nurse sharks sampled. Percentages are calculated based on overall relative abundance across all nurse shark teeth samples.


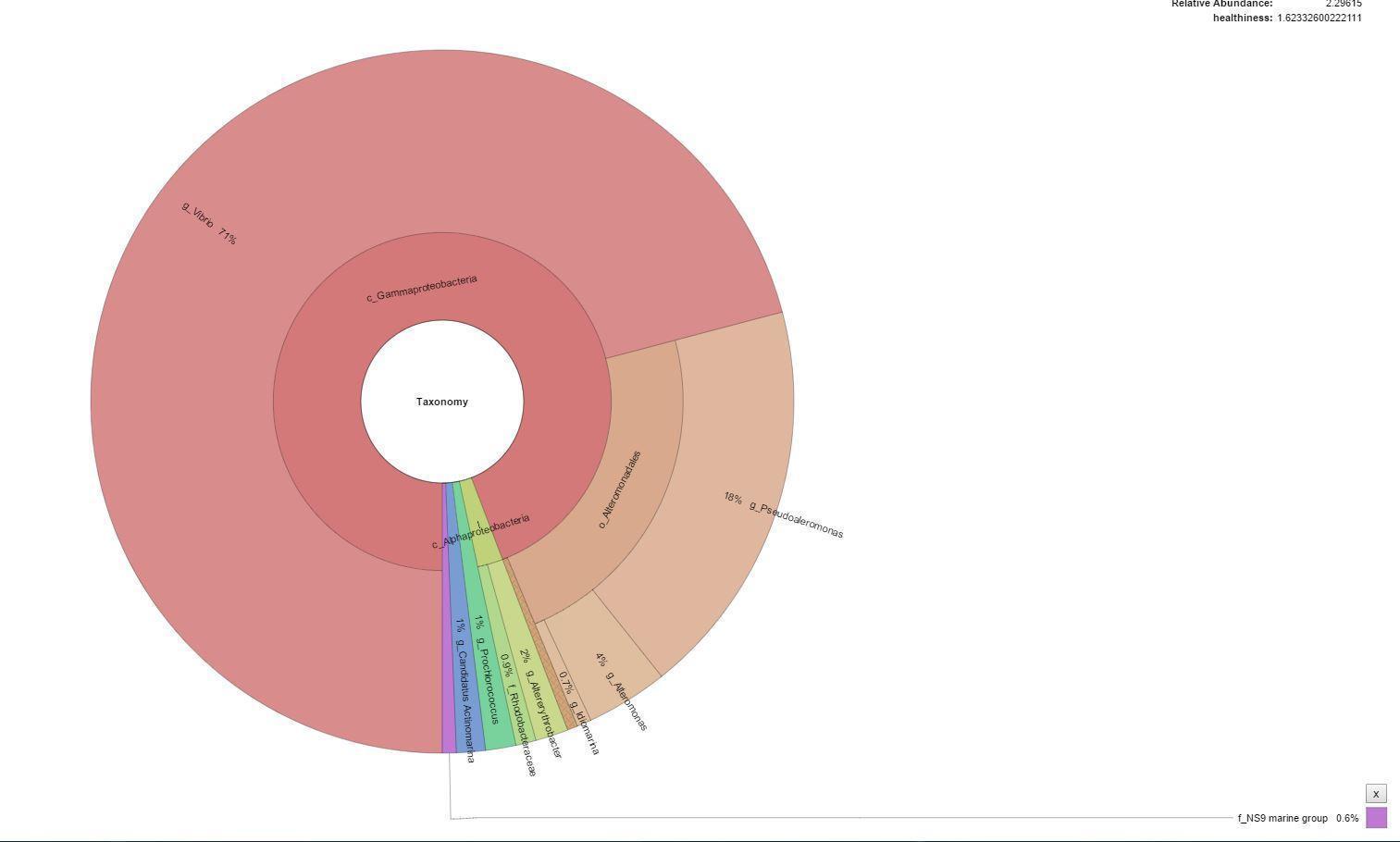


**Figure S12.**  Krona Graphical representation of the most abundant taxa in the microbial community of the teeth of the sandbar sharks sampled. Percentages are calculated based on overall relative abundance across all sandbar shark teeth samples. (Purple=f_NS9 marine group, .6%)


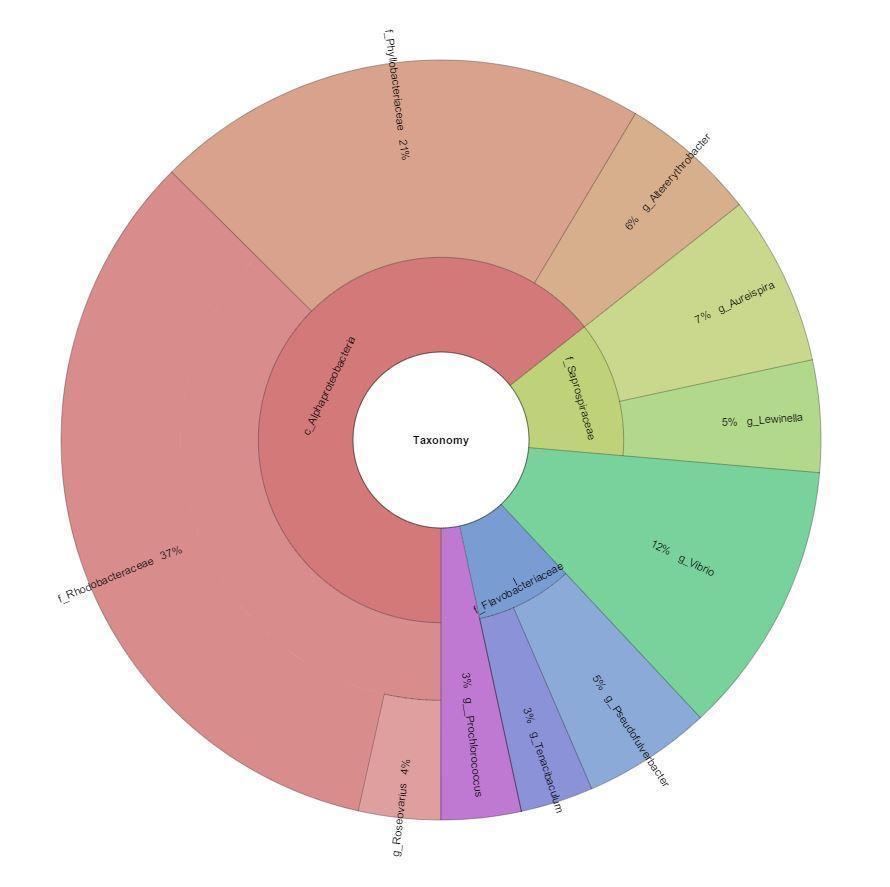


**Figure S13.**  Krona Graphical representation of the most abundant taxa in the microbial community of the teeth of the tiger sharks sampled. Percentages are calculated based on overall relative abundance across all tiger shark teeth samples.

**
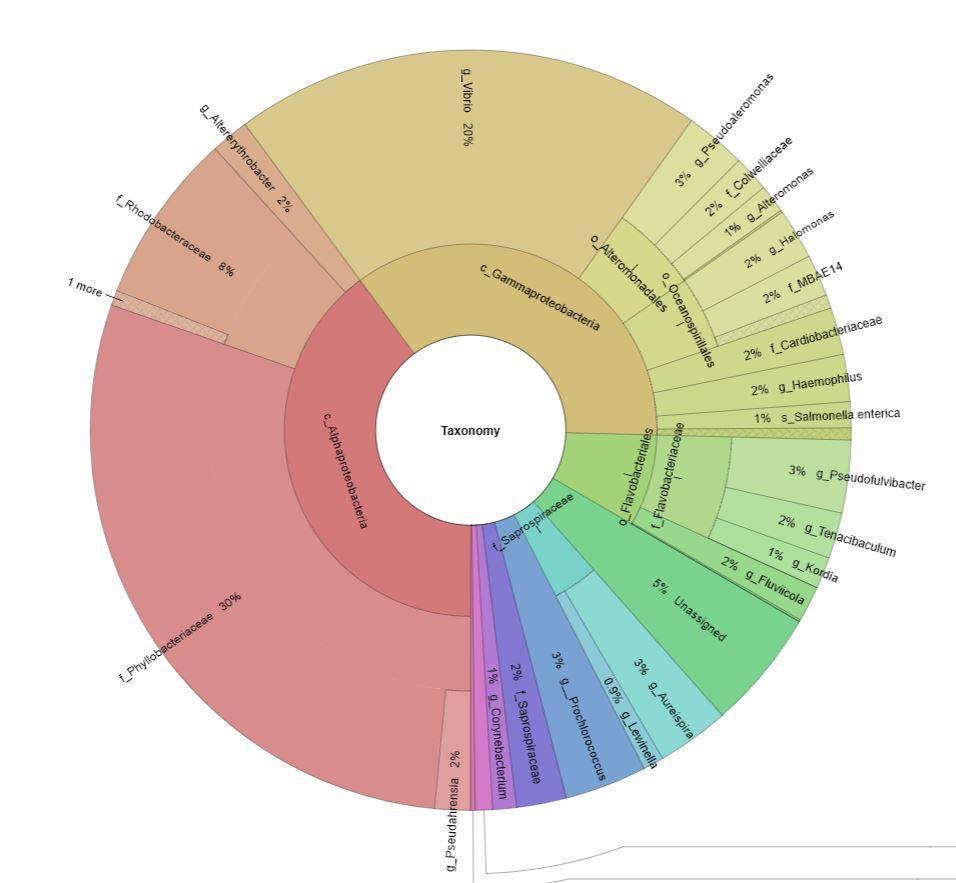
**

**Figure S14.**  Krona Graphical representation of the most abundant taxa in the microbial community of the teeth of sharks sampled. Percentages are calculated based on overall relative abundance across all sandbar shark teeth samples. (Purple=g_*Streptococcus*, .7%, Pink=G_*Canditatus Actinomarina*, .2%)

**
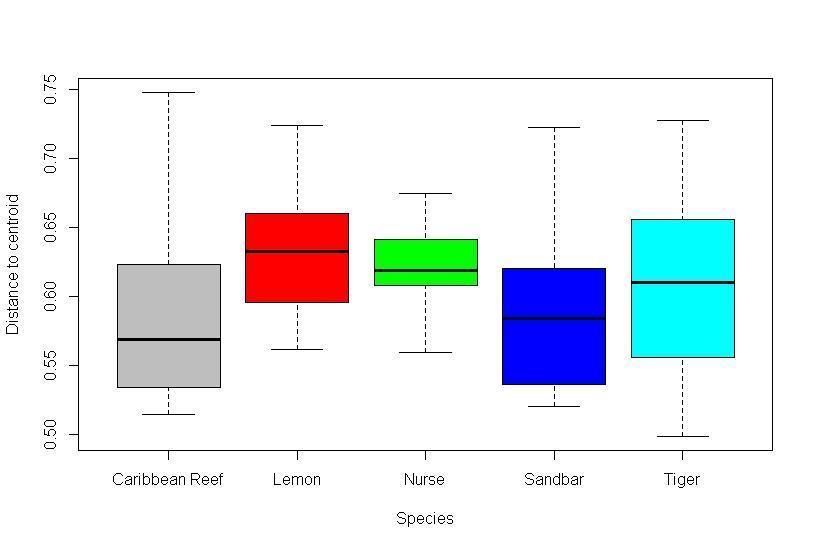
**

**Figure S15.** Beta dispersion analysis of shark teeth samples by species. (ANOVA, df=4, F=3.774, p=.006)


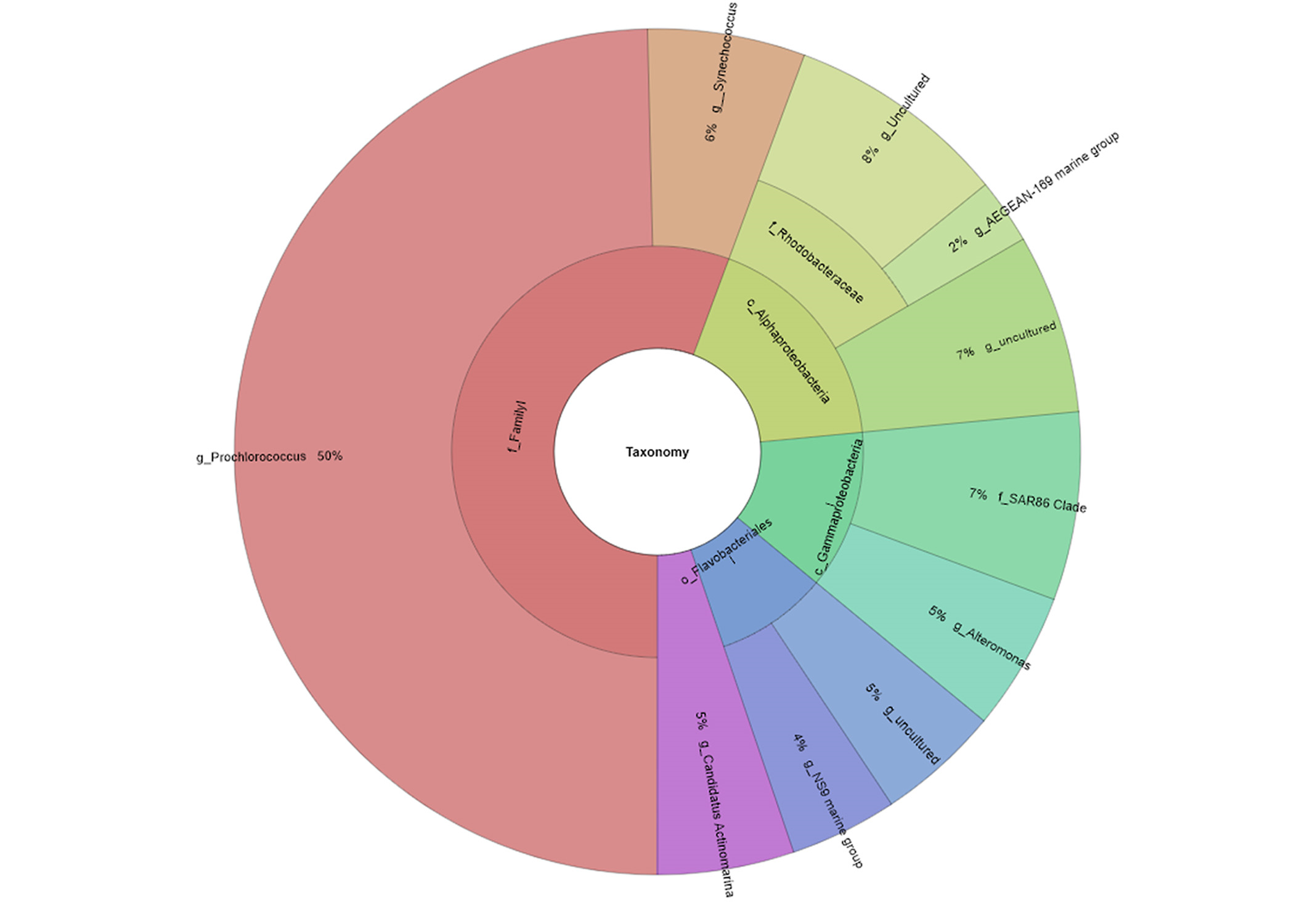


**Figure S16.**  Krona Graphical representation of the most abundant taxa in the microbial community of the water sampled. Percentages are calculated based on overall relative abundance across all water samples.

**Tables**

| Summary Sequencing Statistics | |
| --- | --- |
| **Total MiSeq Reads:** | 12,374,571 |
| **Range for Individual Samples:** | 1,116-545,115 |
| Total OTUs: | 26,309 |

**Table 1.** Summary sequencing data for all species (nurse, tiger, sandbar, Caribbean reef, and lemon) and water samples. Total reads are a sum of all reads for three separate sequencing runs that the samples were sequenced on. Range for individual species indicates the lowest number of reads among all samples, and the highest among all samples.

| **Test** | **P-value** |
| --- | --- |
| Richness | .0377 |
| Diversity-Shannon | .00134 |
| Diversity-Inverse Simpson | .00364 |
| Adonis | .001 |

**Table 2.** Summary of p-values of statistics when teeth samples were compared based on shark species, with environmental OTUs removed.

| **Comparison** | **Sample Size** | **Mean Richness** | **Shannon (ANOVA)** | **Inverse Simpson** |
| --- | --- | --- | --- | --- |
| Species | 117 | 765.6± 516.9 | df=4, F=.512, p=0.727 | df=4, F=1.58, p=0.184 |
| Sample Location | 117 | 765.6± 516.9 | df=3, F=9.832, p<0.001 | df=3, F=4.952, p=0.0029 |
| Species:Location | 117 | 765.6± 516.9 | df=12, F=4.05, p=<0.001 | df=12, F=1.764, p=0.065 |

**Table 3.** Summary of statistics and sample size for each grouping considered in comparisons of the shark microbiome.

| Sample | Species | Sample Location | Month | Type | Latitude | Longitude | Gender |
| --- | --- | --- | --- | --- | --- | --- | --- |
| B051316 | Bait | Bait | May | Environment | 26.09582 | 80.04554 |  |
| B051816 | Bait | Bait | May | Environment | 26.14134 | 80.04847 |  |
| B052416 | Bait | Bait | May | Environment | 26.13789 | 80.039 |  |
| B052616 | Bait | Bait | May | Environment | 26.21083 | 80.03233 |  |
| B060116 | Bait | Bait | June | Environment | 26.09485 | 80.04416 |  |
| B060316 | Bait | Bait | June | Environment | 25.58992 | 80.05762 |  |
| B062216 | Bait | Bait | June | Environment | 26.03651 | 80.05088 |  |
| B063016 | Bait | Bait | June | Environment | 26.03651 | 80.05088 |  |
| B091716 | Bait | Bait | Sept | Environment | 26.12633 | 80.04755 |  |
| B092216 | Bait | Bait | Sept | Environment | 26.18271 | 80.04049 |  |
| B092316 | Bait | Bait | Sept | Environment | 26.00659 | 80.05802 |  |
| B111116 | Bait | Bait | Nov | Environment | 26.02991 | 80.0556 |  |
| C236C | Caribbean Reef | Cloaca | Sept | Shark | 26.18271 | 80.04049 | F |
| C236G | Caribbean Reef | Gills | Sept | Shark | 26.18271 | 80.04049 | F |
| C236S | Caribbean Reef | Skin | Sept | Shark | 26.18271 | 80.04049 | F |
| C236T | Caribbean Reef | Teeth | Sept | Shark | 26.18271 | 80.04049 | F |
| C236W | Water | Water | Sept | Environment | 26.18271 | 80.04049 | F |
| C247C | Caribbean Reef | Cloaca | Nov | Shark | 26.00662 | 80.05114 | F |
| C247G | Caribbean Reef | Gills | Nov | Shark | 26.00662 | 80.05114 | F |
| C247S | Caribbean Reef | Skin | Nov | Shark | 26.00662 | 80.05114 | F |
| C247T | Caribbean Reef | Teeth | Nov | Shark | 26.00662 | 80.05114 | F |
| CR202C | Caribbean Reef | Cloaca | June | Shark | 26.09485 | 80.04416 | F |
| CR202G | Caribbean Reef | Gills | June | Shark | 26.09485 | 80.04416 | F |
| CR202S | Caribbean Reef | Skin | June | Shark | 26.09485 | 80.04416 | F |
| CR202T | Caribbean Reef | Teeth | June | Shark | 26.09485 | 80.04416 | F |
| CR202W | Water | Water | June | Environment | 26.09485 | 80.04416 |  |
| L079C | Lemon | Cloaca | May | Shark | 26.13549 | 80.04914 | F |
| L079G | Lemon | Gills | May | Shark | 26.13549 | 80.04914 | F |
| L079S | Lemon | Skin | May | Shark | 26.13549 | 80.04914 | F |
| L079T | Lemon | Teeth | May | Shark | 26.13549 | 80.04914 | F |
| L079W | Water | Water | May | Environment | 26.13549 | 80.04914 |  |
| L110C | Lemon | Cloaca | May | Shark | 26.09582 | 80.04554 | M |
| L110G | Lemon | Gills | May | Shark | 26.09582 | 80.04554 | M |
| L110S | Lemon | Skin | May | Shark | 26.09582 | 80.04554 | M |
| L110T | Lemon | Teeth | May | Shark | 26.09582 | 80.04554 | M |
| L110W | Water | Water | May | Environment | 26.09582 | 80.04554 |  |
| L164C | Lemon | Cloaca | Sept | Shark | 26.00399 | 80.05626 | M |
| L164G | Lemon | Gills | Sept | Shark | 26.00399 | 80.05626 | M |
| L164S | Lemon | Skin | Sept | Shark | 26.00399 | 80.05626 | M |
| L164T | Lemon | Teeth | Sept | Shark | 26.00399 | 80.05626 | M |
| L164W | Water | Water | Sept | Environment | 26.00399 | 80.05626 |  |
| L169C | Lemon | Cloaca | Sept | Shark | 26.00429 | 80.05195 | F |
| L169G | Lemon | Gills | Sept | Shark | 26.00429 | 80.05195 | F |
| L169S | Lemon | Skin | Sept | Shark | 26.00429 | 80.05195 | F |
| L169T | Lemon | Teeth | Sept | Shark | 26.00429 | 80.05195 | F |
| L169W | Water | Water | Sept | Environment | 26.00429 | 80.05195 | M |
| L191C | Lemon | Cloaca | June | Shark | 26.03369 | 80.05599 | M |
| L191G | Lemon | Gills | June | Shark | 26.03369 | 80.05599 | M |
| L191S | Lemon | Skin | June | Shark | 26.03369 | 80.05599 | M |
| L191T | Lemon | Teeth | June | Shark | 26.03369 | 80.05599 | M |
| L191W | Water | Water | June | Environment | 26.03369 | 80.05599 | M |
| L221C | Lemon | Cloaca | May | Shark | 26.22286 | 80.03008 | M |
| L221G | Lemon | Gills | May | Shark | 26.22286 | 80.03008 | M |
| L221S | Lemon | Skin | May | Shark | 26.22286 | 80.03008 | M |
| L221T | Lemon | Teeth | May | Shark | 26.22286 | 80.03008 | M |
| L221W | Water | Water | May | Environment | 26.22286 | 80.03008 |  |
| L223.W | Water | Water | June | Environment | 26.09695 | 80.0447 |  |
| L223C | Lemon | Cloaca | June | Shark | 26.09695 | 80.0447 | F |
| L223G | Lemon | Gills | June | Shark | 26.09695 | 80.0447 | F |
| L223S | Lemon | Skin | June | Shark | 26.09695 | 80.0447 | F |
| L223T | Lemon | Teeth | June | Shark | 26.09695 | 80.0447 | F |
| L224C | Lemon | Cloaca | June | Shark | 25.58992 | 80.05762 | M |
| L224G | Lemon | Gills | June | Shark | 25.58992 | 80.05762 | M |
| L224S | Lemon | Skin | June | Shark | 25.58992 | 80.05762 | M |
| L224T | Lemon | Teeth | June | Shark | 25.58992 | 80.05762 | M |
| L224W | Water | Water | June | Environment | 25.58992 | 80.05762 |  |
| L225C | Lemon | Cloaca | May | Shark | 26.13789 | 80.039 | M |
| L225G | Lemon | Gills | May | Shark | 26.13789 | 80.039 | M |
| L225S | Lemon | Skin | May | Shark | 26.13789 | 80.039 | M |
| L225T | Lemon | Teeth | May | Shark | 26.13789 | 80.039 | M |
| L225W | Water | Water | May | Environment | 26.13789 | 80.039 |  |
| L231C | Lemon | Cloaca | Sept | Shark | 26.1303 | 80.04121 | F |
| L231G | Lemon | Gills | Sept | Shark | 26.1303 | 80.04121 | F |
| L231S | Lemon | Skin | Sept | Shark | 26.1303 | 80.04121 | F |
| L231T | Lemon | Teeth | Sept | Shark | 26.1303 | 80.04121 | F |
| L231W | Water | Water | Sept | Environment | 26.1303 | 80.04121 |  |
| L238C | Lemon | Cloaca | Sept | Shark | 26.00765 | 80.05163 | M |
| L238G | Lemon | Gills | Sept | Shark | 26.00765 | 80.05163 | M |
| L238S | Lemon | Skin | Sept | Shark | 26.00765 | 80.05163 | M |
| L238T | Lemon | Teeth | Sept | Shark | 26.00765 | 80.05163 | M |
| L238W | Water | Water | Sept | Environment | 26.00765 | 80.05163 |  |
| N080C | Nurse | Cloaca | April | Shark | 26.13353 | 80.08902 | M |
| N080G | Nurse | Gills | April | Shark | 26.13353 | 80.08902 | M |
| N080S | Nurse | Skin | April | Shark | 26.13353 | 80.08902 | M |
| N080T | Nurse | Teeth | April | Shark | 26.13353 | 80.08902 | M |
| N082C | Nurse | Cloaca | April | Shark | 26.13642 | 80.08358 | F |
| N082G | Nurse | Gills | April | Shark | 26.13642 | 80.08358 | F |
| N082S | Nurse | Skin | April | Shark | 26.13642 | 80.08358 | F |
| N082T | Nurse | Teeth | April | Shark | 26.13642 | 80.08358 | F |
| N113C | Nurse | Cloaca | April | Shark | 26.05427 | 80.09445 | F |
| N113G | Nurse | Gills | April | Shark | 26.05427 | 80.09445 | F |
| N113S | Nurse | Skin | April | Shark | 26.05427 | 80.09445 | F |
| N113T | Nurse | Teeth | April | Shark | 26.05427 | 80.09445 | F |
| N114G | Nurse | Gills | April | Shark | 26.36457 | 80.06137 | F |
| N114S | Nurse | Skin | April | Shark | 26.36457 | 80.06137 | F |
| N114T | Nurse | Teeth | April | Shark | 26.36457 | 80.06137 | F |
| N157C | Nurse | Cloaca | May | Shark | 26.02676 | 80.06018 | F |
| N157G | Nurse | Gills | May | Shark | 26.02676 | 80.06018 | F |
| N157S | Nurse | Skin | May | Shark | 26.02676 | 80.06018 | F |
| N157T | Nurse | Teeth | May | Shark | 26.02676 | 80.06018 | F |
| N197C | Nurse | Cloaca | June | Shark | 26.03651 | 80.05088 | M |
| N197G | Nurse | Gills | June | Shark | 26.03651 | 80.05088 | M |
| N197S | Nurse | Skin | June | Shark | 26.03651 | 80.05088 | M |
| N197T | Nurse | Teeth | June | Shark | 26.03651 | 80.05088 | M |
| N197W | Water | Water | June | Environment | 26.03651 | 80.05088 |  |
| N203C | Nurse | Cloaca | June | Shark | 26.03651 | 80.05088 | F |
| N203G | Nurse | Gills | June | Shark | 26.03651 | 80.05088 | F |
| N203S | Nurse | Skin | June | Shark | 26.03651 | 80.05088 | F |
| N203T | Nurse | Teeth | June | Shark | 26.03651 | 80.05088 | F |
| N203W | Water | Water | June | Environment | 26.03651 | 80.05088 |  |
| N235C | Nurse | Cloaca | Sept | Shark | 26.13051 | 80.0434 | M |
| N235G | Nurse | Gills | Sept | Shark | 26.13051 | 80.0434 | M |
| N235S | Nurse | Skin | Sept | Shark | 26.13051 | 80.0434 | M |
| N235T | Nurse | Teeth | Sept | Shark | 26.13051 | 80.0434 | M |
| N235W | Water | Water | Sept | Environment | 26.13051 | 80.0434 |  |
| SB159C | Sandbar | Cloaca | May | Shark | 26.09699 | 80.04415 | F |
| SB159G | Sandbar | Gills | May | Shark | 26.09699 | 80.04415 | F |
| SB159S | Sandbar | Skin | May | Shark | 26.09699 | 80.04415 | F |
| SB159T | Sandbar | Teeth | May | Shark | 26.09699 | 80.04415 | F |
| SB159W | Water | Water | May | Environment | 26.09699 | 80.04415 |  |
| SB174C | Sandbar | Cloaca | May | Shark | 26.07791 | 80.05269 | F |
| SB174G | Sandbar | Gills | May | Shark | 26.07791 | 80.05269 | F |
| SB174S | Sandbar | Skin | May | Shark | 26.07791 | 80.05269 | F |
| SB174T | Sandbar | Teeth | May | Shark | 26.07791 | 80.05269 | F |
| SB174W | Water | Water | May | Environment | 26.07791 | 80.05269 |  |
| SB198C | Sandbar | Cloaca | June | Shark | 25.58854 | 80.05157 | F |
| SB198G | Sandbar | Gills | June | Shark | 25.58854 | 80.05157 | F |
| SB198S | Sandbar | Skin | June | Shark | 25.58854 | 80.05157 | F |
| SB198T | Sandbar | Teeth | June | Shark | 25.58854 | 80.05157 | F |
| SB198W | Water | Water | June | Environment | 25.58854 | 80.05157 |  |
| T209C | Tiger | Cloaca | May | Shark | 26.20859 | 80.0344 | F |
| T209G | Tiger | Gills | May | Shark | 26.20859 | 80.0344 | F |
| T209S | Tiger | Skin | May | Shark | 26.20859 | 80.0344 | F |
| T209T | Tiger | Teeth | May | Shark | 26.20859 | 80.0344 | F |
| T209W | Water | Water | May | Environment | 26.20859 | 80.0344 |  |
| T228C | Tiger | Cloaca | Sept | Shark | 26.00659 | 80.05802 | F |
| T228G | Tiger | Gills | Sept | Shark | 26.00659 | 80.05802 | F |
| T228S | Tiger | Skin | Sept | Shark | 26.00659 | 80.05802 | F |
| T228T | Tiger | Teeth | Sept | Shark | 26.00659 | 80.05802 | F |
| T228W | Water | Water | Sept | Environment | 26.00659 | 80.05802 |  |
| TGH107C | Tiger | Cloaca | May | Shark | 26.09456 | 80.05056 | F |
| TGH107G | Tiger | Gills | May | Shark | 26.09456 | 80.05056 | F |
| TGH107S | Tiger | Skin | May | Shark | 26.09456 | 80.05056 | F |
| TGH107T | Tiger | Teeth | May | Shark | 26.09456 | 80.05056 | F |
| TGH116C | Tiger | Cloaca | Nov | Shark | 26.02914 | 80.05036 | F |
| TGH116G | Tiger | Gills | Nov | Shark | 26.02914 | 80.05036 | F |
| TGH116S | Tiger | Skin | Nov | Shark | 26.02914 | 80.05036 | F |
| TGH116T | Tiger | Teeth | Nov | Shark | 26.02914 | 80.05036 | F |
| TGH117C | Tiger | Cloaca | Nov | Shark | 26.08349 | 80.04573 | F |
| TGH117G | Tiger | Gills | Nov | Shark | 26.08349 | 80.04573 | F |
| TGH117S | Tiger | Skin | Nov | Shark | 26.08349 | 80.04573 | F |
| TGH117T | Tiger | Teeth | Nov | Shark | 26.08349 | 80.04573 | F |
| TGH117W | Water | Water | Nov | Environment | 26.08349 | 80.04573 |  |
| TGH240C | Tiger | Cloaca | Nov | Shark | 26.08018 | 80.05205 | M |
| TGH240G | Tiger | Gills | Nov | Shark | 26.08018 | 80.05205 | M |
| TGH240S | Tiger | Skin | Nov | Shark | 26.08018 | 80.05205 | M |
| TGH240T | Tiger | Teeth | Nov | Shark | 26.08018 | 80.05205 | M |

**Table 4.** Sample table summarizing all environmental and shark samples
